# The role of platelets in cancer: from their influence on tumor progression to their potential use in liquid biopsy

**DOI:** 10.1186/s40364-025-00742-w

**Published:** 2025-02-11

**Authors:** Miguel Morales-Pacheco, Miguel Valenzuela-Mayen, Angel M. Gonzalez-Alatriste, Gretel Mendoza-Almanza, Sergio A. Cortés-Ramírez, Alberto Losada-García, Griselda Rodríguez-Martínez, Imelda González-Ramírez, Vilma Maldonado-Lagunas, Karla Vazquez-Santillan, Vanessa González-Covarrubias, Carlos Pérez-Plasencia, Mauricio Rodríguez-Dorantes

**Affiliations:** 1 Laboratorio de Oncogenómica, Instituto Nacional de Medicina Genómica, Mexico City, 14610 Mexico; 2 Laboratorio de Epigenética, Instituto Nacional de Medicina Genómica, Secretaría de Salud, Mexico City, 14610 Mexico; 3 Department of Pharmacology and Toxicology, Winthrop P. Rockefeller Cancer Institute, University of Arkansas for Medical Sciences, Little Rock, AR 72205 USA; 4 Laboratorio de Investigación en Patógenos Respiratorios y Producción de Biológicos, Hospital Infantil de México Federico Gómez, Mexico City, 14610 Mexico; 5 Departamento de Atención a La Salud, Universidad Autónoma Metropolitana Xochimilco, Mexico City, 14610 Mexico; 6 Laboratorio de Innovación en Medicina de Precisión, Instituto Nacional de Medicina Genómica, Secretaría de Salud, Mexico City, 14610 Mexico; 7 Laboratorio de Farmacogenómica, Instituto Nacional de Medicina Genómica, Secretaría de Salud, Mexico City, 14610 Mexico; 8 Laboratorio de Genómica, FES-Iztacala, Universidad Nacional Autónoma de México (UNAM), Iztacala, Tlalnepantla 54090 Mexico

**Keywords:** Cancer, Tumor metastasis, Platelets, Tumor-educated platelets, Biomarkers, Liquid biopsy

## Abstract

Platelets, anucleate blood cells essential for hemostasis, are increasingly recognized for their role in cancer, challenging the traditional notion of their sole involvement in blood coagulation. It has been demonstrated that platelets establish bidirectional communication with tumor cells, contributing to tumor progression and metastasis through diverse molecular mechanisms such as modulation of proliferation, angiogenesis, epithelial-mesenchymal transition, resistance to anoikis, immune evasion, extravasation, chemoresistance, among other processes. Reciprocally, cancer significantly alters platelets in their count and composition, including mRNA, non-coding RNA, proteins, and lipids, product of both internal synthesis and the uptake of tumor-derived molecules. This phenomenon gives rise to tumor-educated platelets (TEPs), which are emerging as promising tools for the development of liquid biopsies. In this review, we provide a detailed overview of the dynamic roles of platelets in tumor development and progression as well as their use in diagnosis and prognosis. We also provide our view on current limitations, challenges and future research areas, including the need to design more efficient strategies for their isolation and analysis, as well as the validation of their sensitivity and specificity through large-scale and rigorous clinical trials. This research will not only enable the evaluation of their clinical viability but could also open new opportunities to enhance diagnostic accuracy and develop personalized treatments in oncology.

## Background

Platelets are small anucleated cells of 2–4 μm in diameter, mostly synthesized in bone marrow by megakaryocytes, with the main function of maintaining hemostasis [1]. Hemostasis is divided into primary and secondary stages; primary hemostasis involves the recruitment and activation of platelets to form a platelet plug. Platelet activation involves several processes, such as platelet shape change, release of their granule contents, and activation of cell surface receptors, such as GP IIb/IIIa, which facilitates platelet adhesion and aggregation at the injury site. This process depends on the interaction with collagen and soluble agonists, such as ADP, thromboxane A2 (TxA2), and thrombin, which amplify platelet activation. Through these pathways, platelets aggregate with each other, forming the platelet plug necessary to stop bleeding. Secondary hemostasis involves the activation of the coagulation system to generate fibrin, thus stabilizing the platelet plug and effectively stopping bleeding [2, 3].

Platelets are recognized as cells with remarkable molecular complexity, which confers them the ability to play crucial roles in a variety of physiological and pathophysiological processes, including a role in cancer. In this review, we focus on the molecular mechanisms through which platelets participate in cancer progression and their clinical and therapeutic implications. Furthermore, due to the remarkable ability of platelets to alter their content in the presence of a tumor, we present evidence for their potential use as liquid biopsies in cancer diagnosis, prognosis, and monitoring, exploring both their benefits and limitations.

### Association between platelets and cancer

The association between cancer and platelets was first described in 1865 by Armand Trousseau, who reported that blood coagulation was a common event in cancer patients [4]. A few years later, in 1878, Billroth expanded on this observation by discovering that metastatic tumors often contained thrombi, suggesting a possible link between coagulation and metastasis [5]. Although platelets had not yet identified, Giulio Bizzozero identified them in 1881, providing key insights into their role in blood coagulation [6]. These early findings laid the groundwork for future discoveries.

It wasn’t until the mid-twentieth century that a direct connection between platelets and cancer metastasis was established. In 1968, Gasic and colleagues used an in vivo model to demostrate that low platelet count (thrombocytopenia) could reduce metastasis, while the induction of platelet-rich plasma (PRP) reversed this effect. This highlighted the crucial role of platelets in cancer progression [7]. Around the same time, Levin and Conley observed that elevated platelet count (thrombocytosis) was often associated with malignant disease [8], further implicating platelets in cancer biology. These studies prompted a growing body of research aimed at understanding the specific mechanisms by which platelets influence tumor progression.

Recent studies have suggested that elevated platelet counts might serve as a biomarker for cancer diagnosis [9–11]. Moreover, thrombocytosis has been consistently linked to poorer survival rates and the presence of metastasis in cancer patients [12]. This correlation has been particularly noted in patients with colorectal [13–16], gastric [17, 18], lung [19, 20], and gynecological cancers [21–27]. However, the precise role of thrombocytosis in cancer progression remains unclear. It is still debated whether an increased platelet count is a risk factor for cancer, a consequence of the disease, or both. Notably, tumor-derived interleukin-6 and hepatic thrombopoietin, key players in thrombopoiesis, are elevated in cancer [28], contributing to the rise of circulating platelets, partly due to an increased megakaryopoiesis [29]. Interestingly, platelet counts have been reported to decrease following the removal of malignant tumors [30], reinforcing the notion that the tumor itself plays a role in driving thrombocytosis. Hence, there is growing evidence that a tumor can stimulate an increase in platelet numbers, which may, in turn, facilitate cancer progression through various mechanisms. In the next section, we explore the molecular mechanisms by which platelets contribute to tumor growth and metastasis.

### Mechanisms of platelet-cancer cell interaction

The tumor microenvironment (TME) surrounding cancer cells includes various cell types, including endothelial cells, fibroblasts, adipocytes, and immune cells. It also includes non-cellular components such as the extracellular matrix, cytokines, growth factors, and extracellular vesicles [31]. However, platelets have also been recognized as an important component of the TME. Significant platelet infiltration and aggregation have been observed in a wide range of cancers such as breast, colorectal, hepatocellular, gastric, and pancreatic [32]. Platelets also interact with circulating tumor cells (CTCs) in the breast [33], melanoma [34], pancreatic [35] and prostate cancer [36, 37]. These observations support the hypothesis that platelets are a crucial element of the TME, playing an active role in tumor cell interactions.

Platelets and tumor cells can interact physically through direct binding between receptors or via extracellular proteins possibly through the expression of glycoproteins. For example, interactions have been observed between platelet GPVI and tumor galectin-3 [38], platelet integrin α6β1 and tumor ADAM9 [39], platelet CLEC-2 and tumor podoplanin [40], and platelet GPIIb/IIIa and tumor ανβ3 [41]. Platelet P-selectin interacts with its ligand on the tumor [42–45]. Additionally, extracellular proteins like fibrinogen [46], fibronectin [47], and von Willebrand factor (VWF) [48] can also mediate these interactions. These complex interactions highlight the intricate relationship between platelets and tumor cells. For example, in animal models, has been shown that blocking certain glycoproteins, such as GPIbα [48, 49], ανβ3 [41], α6β1 [39], and GPVI [38], reduces metastatic progression, suggesting that targeting these proteins could be a promising strategy for cancer therapy.

Moreover, the interaction between platelets and tumor cells triggers a phenomenon known as tumor cell-induced platelet aggregation (TCIPA). To induce TCIPA within the TME, cancer cells can express molecules or agonists that interact with platelet receptors, initiating platelet activation. Once activated, platelets release granules containing additional platelet agonists, which amplify the activation response. This release not only enhances platelet activation but also contributes to tumor growth and the spread of metastasis [50].

### Influence of platelet-derived microparticles on cancer cells

The interaction between platelets and tumor cells can also occur through extracellular vesicles. Under physiological conditions or following an activating stimulus such as TCIPA, platelets release microvesicles, also known as platelet-derived microparticles (PMPs) and exosomes. The composition of these extracellular vesicles varies depending on the stimulus, resulting in highly dynamic and versatile populations [51]. PMPs have been identified as key regulators in intracellular communication, and their role in cancer has attracted considerable attention. In several types of cancer, including melanoma [52, 53], lung [54], breast [55], prostate [56], oral [57], colorectal [58] and liver [59], it has been observed an increment of PMPs in plasma, correlated with increased platelet activity induced by tumor cells. Since PMPs are increased in cancer and have the ability to internalize into tumor cells, they can deliver bioactive contents such as proteins, nucleic acids, signaling molecules, membrane receptors, lipids, and even mitochondria. These components act on the recipient cells and can modify the intercellular signaling cascade of the recipient cell, potentially favoring cancer progression [60, 61].

Additionally, tumor cell-derived microvesicles have also been associated with TCIPA [62]. Once released by the tumor, these microvesicles can interact with platelets inducing activation and aggregation, thus contributing to tumor progression and cancer dissemination. This interaction establishes bidirectional communication between tumor cells and platelets, either through direct or indirect contact, the release of agonists, microvesicles, or PMPs, ultimately promoting tumor growth and facilitating metastatic expansion.

### Platelets promote the proliferation of tumor cells

Cancer cells are characterized by their ability to proliferate continuously. Unlike normal tissues, which regulate the production and release of signals that promote cell growth to maintain homeostasis and normal tissue function, cancer cells deregulate these signals, acquiring autonomous control over their fate [63].

It is widely documented that platelets serve as an important reservoir of growth factors, including platelet-derived growth factor (PDGF), vascular endothelial growth factor (VEGF), transforming growth factor (TGF), insulin-like growth factor binding protein (IGFBP), interleukins (ILs), fibroblast growth factor (FGF), platelet-derived angiogenesis factor (PDAF), epidermal growth factor (EGF), and insulin-like growth factor (IGF), among others [64, 65]. These growth factors are crucial for tissue repair and wound healing. Recently, platelets and their extracts have been proposed as treatments for wound healing [66, 67]. In cancer, these growth factors can promote tumor growth. In fact, studies have shown that the release of platelet contents can induce cell proliferation. In various types of cancer such as breast [68, 69], colorectal [70], osteosarcoma [71], hepatocellular [71], gastric [72] and ovarian cancer [73], platelets have been shown to promote cell proliferation. In ovarian cancer cells specifically, the release of platelet-derived TGF-β was found to stimulate cell proliferation. Conversely, blocking TGF-β with an antibody or silencing the TGF-β receptor suppressed this effect [73]. In liver cancer, platelet-derived TGF-β was observed to inhibit the expression of the tumor suppressor Krüppel-like factor 6 (KLF6) [74].

Moreover, by releasing growth factors, platelets also influence cell signaling pathways involved in cancer. Studies with osteosarcoma cultured cells with platelets showed an increased cell proliferation due to the activation of the PDGFR-Akt signaling axis [71]. Similarly, in colon cancer cells, platelets facilitated tumor growth by upregulating and activating the oncoprotein c-MYC [70]. Nevertheless, some studies have reported opposing observations suggesting that platelets might alter the cell cycle inhibiting cell proliferation [75].

**Fig. 1:**
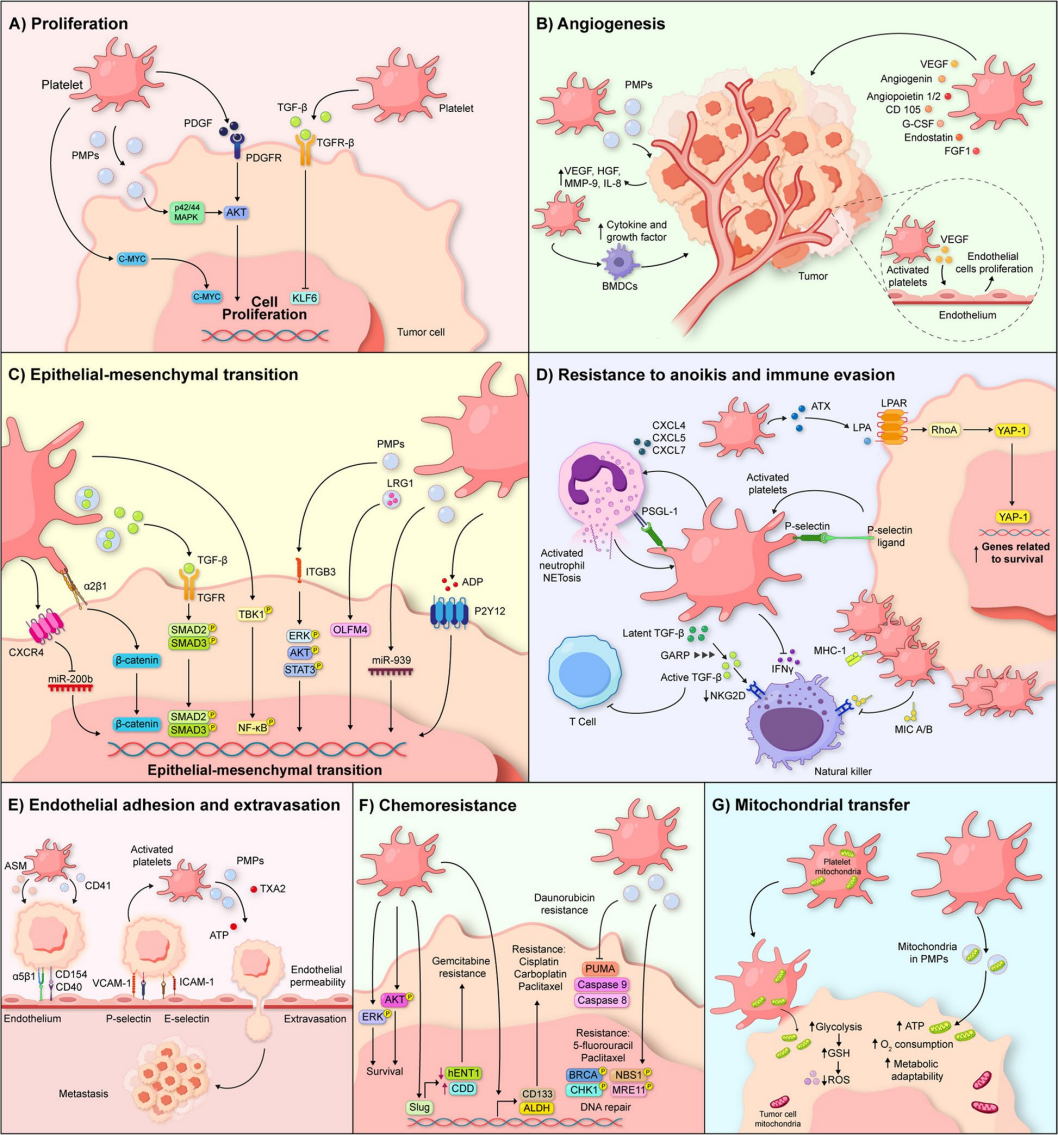
Schematic representation of the different mechanisms by which platelets promote cancer progression. **A** Platelets promote cell proliferation by releasing growth factors such as PDGF and TGF-β, which activate signaling pathways. Platelet microvesicles (PMPs) can also induce cell proliferation. **B** Platelets secrete proangiogenic factors into the tumor microenvironment (TME), promoting the formation of new blood vessels. Platelets can be activated by endothelial cells, which favors the secretion of more proangiogenic factors, such as VEGF and other key components that contribute to the growth and maturation of blood vessels in tumors. **C** Platelets also contribute to epithelial-mesenchymal transition (EMT), a process that allows tumor cells to migrate and invade tissues during metastasis. Upon infiltrating the TME, platelets release TGF-β, a factor that induces phenotypic changes in tumor cells, increasing their ability to move and invade. Platelet activation, mediated by interactions with tumor cells, is also related to the activation of various signaling pathways, such as TGF-β/Smad and Wnt-β-catenin, which are crucial for EMT. **D** Platelets interact with circulating tumor cells (CTCs) through the release of signaling molecules and direct contact. This interaction activates platelets and promotes the activation of the RhoA/YAP-1 pathway in CTCs, inducing resistance to anoikis and allowing these cells to survive and disseminate in the bloodstream. Furthermore, platelets contribute to immune evasion by releasing TGF-β and MICA/MICB, which inhibit natural killer (NK) and T cell activity, and by recruiting neutrophils to metastatic sites. **E** Activated platelets form complexes with CTCs through P-selectin and integrins, facilitating their adhesion to the endothelium. Platelets subsequently proteolyze the extracellular matrix and release molecules that increase endothelial permeability, facilitating CTCs extravasation. **F** Platelets promote chemoresistance by promoting cell survival, inhibiting apoptosis, regulating drug metabolism, and activating DNA repair mechanisms. **G** In the TME, platelets transfer mitochondria to tumor cells by various mechanisms, including the release of microparticles. These microparticles, which contain mitochondria, are internalized by tumor cells, leading to metabolic reprogramming and increased malignancy

On the other hand, in leukemia cells, PMPs internalization has been shown to enhance tumor cell growth [76], but in lung cells, PMPs internalization induces cell proliferation through the activation of the MAPK p42/44 and AKT signaling pathways [77] (Fig. 1A). Interestingly, in colon cancer cells, PMPs internalization does not affect cell proliferation [60], suggesting that the impact of PMPs may vary depending on the type of cancer, and hence the micro and macro environment.

Taken together, these studies suggest that platelets play a multifaceted role in cancer cell proliferation by releasing growth factors and modifying cell signaling pathways. Understanding these interactions is crucial for developing more effective cancer therapies.

### Platelets are involved in angiogenesis

Angiogenesis, the formation of new blood vessels, is a complex process regulated by pro- and anti-angiogenic molecules. Under physiological conditions, this process supports embryonic development, tissue repair, the menstrual cycle, muscle growth, and organ regeneration. However, in pathological conditions such as cancer, angiogenesis becomes a critical mechanism that supplies oxygen and nutrients to cancer cells, enhancing their survival and promoting tumor progression [78]. Angiogenesis is essential not only for the intravasation, extravasation, and growth of malignant cells at distant sites but also for the development of the TME.

Tumor-driven angiogenesis involves contributions from cancer cells and other cellular components, including stromal cells, tumor-associated macrophages, endothelial cells, and platelets, which release proangiogenic factors to sustain this process [79].

Platelets are well-known to contain proangiogenic factors, and studies have demonstrated their role in facilitating angiogenesis both in vitro [80] and in vivo [81]. When activated by endothelial cells, platelets adhere to these cells and stimulate their proliferation by releasing VEGF [82]. Specifically, activated platelets in the TME contribute to elevated concentrations of vascular endothelial growth factor (VEGF) and other proangiogenic components, including angiogenin, angiopoietin 1 and 2, G-CSF, CD 105, FGF 1, and endostatin [83]. Increased expression of angiogenesis and endothelial activation markers, such as VEGF, VWF, P-selectin, VEGFR2, and S1P (Sphingosine-1-phosphate), is also observed in platelets from cancer patients [84].

Furthermore, bone marrow-derived cells (BMDCs) contribute to blood vessel maturation by secreting growth factors and cytokines within tumors. BMDCs are recruited to the TME, and subsequently, the release of platelet α-granules in response to hypoxia promotes BMDC recruitment, underscoring the crucial role of platelets in tumor angiogenesis [85]. Platelet-derived extracellular vesicles (PEVs) are also implicated in this process, as they enhance the expression of MMP-9, VEGF, HGF, and IL-8 in tumor cells, further highlighting the importance of platelets in regulating angiogenesis in the tumor context [77] (Fig. 1B). Understanding how platelets promote angiogenesis may provide new insights for developing targeted therapies to modulate tumor angiogenesis and improve cancer treatment approaches.

### Platelets promote epithelial-mesenchymal transition, migration and invasion of tumor cells

Metastasis begins with primary invasion, where tumor cells actively migrate from their site of origin through the extracellular matrix to blood vessels or lymph nodes [86]. Epithelial-mesenchymal transition (EMT) is a critical early step in metastasis for several types of cancer, transforming epithelial cells into mesenchymal cells, and increasing their mobility and invasive capacity. During EMT, cells lose their polarity and cell–cell adhesion features, undergoing cytoskeletal modifications [87]. At the molecular level, it involves reduced expression of epithelial markers (e.g., E-cadherin) and increased expression of mesenchymal markers (e.g., vimentin and N-cadherin), often correlating with alterations in cell morphology. Within TME, interactions between tumor cells and other cellular components—such as macrophages, fibroblasts, cancer stem cells, endothelial cells, lymphocytes, and platelets—along with soluble factors, significantly influence EMT [88]. Platelets play an essential role in promoting EMT through several signaling pathways and cell interaction mechanisms. They infiltrate the TME via focal adhesion kinase (FAK) and tissue factor [89]. In tumor tissues, the platelet activation marker CD42b has been observed to colocalize with EMT markers, suggesting the key role of activated platelets in EMT [90]. In addition, platelets can induce partial EMT in CTCs, a process known as epithelial-mesenchymal plasticity [91]. This process highlights the ability of tumoral cells to adapt and survive in the circulation and during metastasis [92].

TGF-β is the most studied factor that can regulate EMT. Platelets are the primary source of circulating TGF-β1, containing 40 to 100 times more than any other cell and can be released upon platelet activation. Interestingly, in patients with ovarian cancer, both platelet count and serum TGF-β levels have been reported to be elevated, suggesting a possible correlation between platelet activity, TGF-β release, and tumor progression [93]. In vitro studies have shown that the incubation of platelets with various types of cancer cells, such as ovarian [93, 94], breast, colon [95], bladder and mesothelioma [96], leads to an increase in the expression of markers associated with EMT. This interaction induces a phenotypic change towards a mesenchymal state and enhances the invasive capacity of these cells. This process is partly mediated by direct contact between platelets and tumor cells, resulting in platelet activation and an increase in TGF-β levels, which in turn activates the TGF-β/Smad signaling pathway.

The effect of TGF-β has also been demonstrated through indirect contact between tumor cells and platelets. In colon cancer, conditioned medium from platelet-activated tumor cells has been shown to induce EMT by activating the TGF-β/Smad pathway [97]. Furthermore, the release of TGF-β1 by activated platelets leads to an upregulation of CXCR4 and inhibition of miR-200b in colon cancer cells, promoting EMT [98]. TGF-β also exerts its effects through exosomes. High levels of TGF-β in platelet-derived exosomes have been linked to increased migration and invasiveness in breast cancer cells [99].

These data suggest that tumor cells can activate platelets, which in turn release soluble or extracellular vesicular TGF-β to induce EMT through various signaling pathways. To support this hypothesis, the addition of TGF-βRI inhibitors, such as SB431542 or A-83–01, or the use of TGF-β1 blocking antibodies, has been shown to abolish platelet-induced EMT [93, 95]. Furthermore, Hu et al. demonstrated that TGF-β1-deficient platelets resulted in a significant reduction in metastatic foci [100]. These observations highlight the central role of TGF-β in regulating platelet-mediated EMT, suggesting that strategies targeting TGF-β could be crucial for preventing metastasis in cancer.

In addition to the TGF-β1 pathway, other signaling pathways can be activated in tumor cells in response to platelets. For example, direct contact between platelets and MCF7 breast cancer cells is mediated by integrin α2β1, which induces both the autocrine TGF-β1/pSmad3 pathway and the Wnt-β-catenin pathway, triggering EMT [101]. In breast cancer, TANK-binding kinase 1 (TBK1) has been identified as a mediator of platelet-induced EMT. Platelets activate TBK1, leading to the activation of the p65 subunit of NF-κB and subsequent EMT induction [102]. Furthermore, platelets induce EMT in a transgenic mouse model of pancreatic cancer (RIP1-Tag2), with an even stronger effect in the absence of histidine-rich glycoprotein (HRG). In this model, activated platelets induce EMT by activating TBK1, Akt2, and PDGFRβ signaling pathways in the TME, resulting in an increased rate of spontaneous metastasis to distant organs, such as the liver, in the absence of HRG. These findings suggest that HRG acts as a tumor suppressor by regulating platelet activity and, consequently, tumor progression [103]. In HepG2 and SMMC-7721 liver cancer cells, both platelet-rich plasma and platelets, as well as platelet-released content, have been shown to activate the MAPK/AKT/STAT3 signaling axis and promote EMT [104].

On the other hand, PMPs and exosomes can internalize into tumor cells. These extracellular vesicles not only facilitate intercellular communication but also play a crucial role in modulating the TME. For example, in ovarian cancer, the internalization of PMPs leads to the release and upregulation of miR-939, which alters the expression of EMT-related genes [105]. In nasopharyngeal cancer [106] and colorectal cancer [107], extracellular vesicles derived from platelets of cancer patients have induced EMT through the upregulation of ITGB3 expression and increased expression of PTGS2 and TXB2, respectively. Likewise, exosomes from platelets in patients with multiple myeloma presented elevated levels of leucine-rich alpha-2-glycoprotein 1 (LRG1), which interacts with Olfactomedin 4 (OLFM4) in tumor cells, activating the EMT signaling pathway [108] (Fig. 1C).

**Fig. 2:**
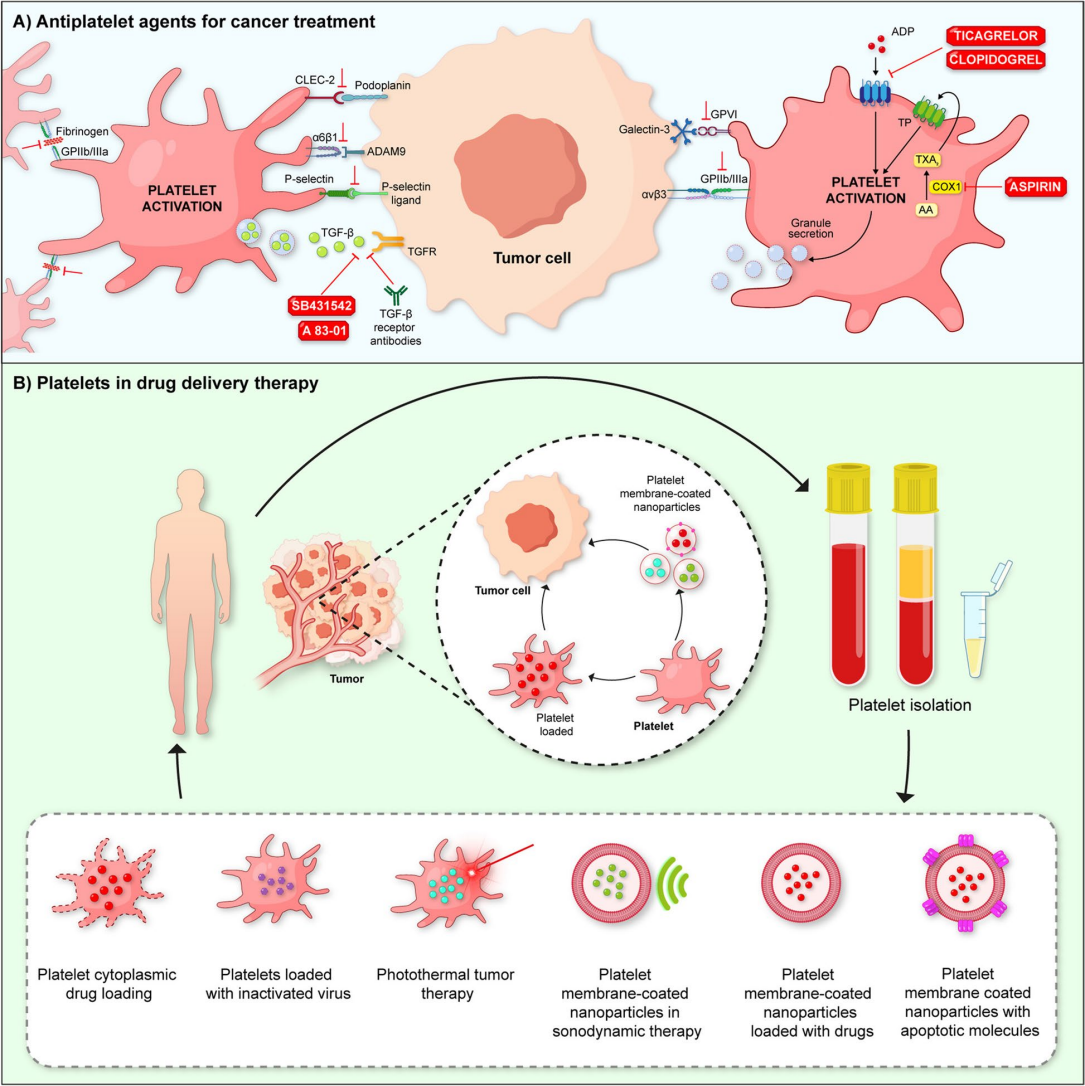
Blocking platelet activity and using platelets as therapeutic vehicles in cancer. **A** The interaction of platelets with tumor cells induces their activation and the release of components that promote tumor progression. P2Y12 receptor inhibitors such as ticagrelor and (Cyclooxygenase-1) COX-1 inhibitors such as aspirin can interfere with platelet aggregation, thus blocking the effect of platelets on cancer. Furthermore, blocking platelet receptors could represent a viable strategy for cancer treatment. **B** Platelets can be therapeutic vehicles in cancer, facilitating the delivery of drugs, such as doxorubicin, to tumor cells. Loading drugs in platelets improves tumor accumulation and reduces side effects. Furthermore, their genetic modification and use in photothermal and nanoparticle therapy expand their potential in targeted cancer treatments

Platelets facilitate EMT through multiple mechanisms, so targeting these specific mechanisms is crucial to halt platelet-associated tumor progression. In particular, inhibition of the P2Y12 receptor has emerged as a promising strategy to modulate platelet-induced EMT. In colon, pancreatic, and ovarian cancers, tumor cells activate and aggregate platelets, promoting the expression of EMT markers and the formation of metastatic foci [109–112]. This process is largely mediated by the release of ADP from platelets, which activates the P2Y12 receptor on tumor cells. Administration of ticagrelor and 2-methylthioadenosine 5′-monophosphate triethylammonium salt hydrate, both P2Y12 receptor inhibitors, has been shown to reduce these effects, underscoring the importance of this pathway in modulating platelet-mediated EMT [112]. Similarly, blocking platelet aggregation with aspirin, a Cyclooxygenase-1 (COX-1) inhibitor, has proven effective in inhibiting platelet-induced EMT. Aspirin works by blocking the formation of thromboxane A2, a pro-platelet aggregating agent, which reduces platelet activation and subsequent EMT induction [104] (Fig. 2A). Thus, although platelets play a crucial role in promoting cancer through multiple pathways, specific inhibition of P2Y12 and the use of other antiplatelet agents may be effective strategies to limit tumor progression and metastasis. These targeted therapies offer a promising approach to controlling cancer spread.

### Platelets interact with circulating tumor cells

The connection between a tumor's primary site and distant metastases is established by CTCs, which disseminate from the tumor, traverse the endothelium, and enter the bloodstream [113]. Upon entering the bloodstream, CTCs face numerous challenges, including hydrodynamic shear stress, high pressure, immune attack by natural killer (NK) cells, oxidative stress, oxygen and nutrient deprivation, and the lack of adherence to a substrate. Consequently, the majority of CTCs fail to survive in circulation, with only a small fraction (approximately 0.1%) overcoming these obstacles to evade the immune system and reach the microvasculature of distant tissues, where they can infiltrate and establish metastatic niches [113–115].

Remarkably, platelets can improve the survival and transit of CTCs within bloodstream. Interestingly, platelets interact with CTCs in two ways: by releasing signaling molecules such as thrombin, ADP, and tumor-associated proteinases or through direct contact. The direct interaction between CTCs and platelets is not well understood due to limited information on platelet receptors in tumors. Evidence suggests that CD97, an adhesion G-protein-coupled receptor overexpressed on most cancer cells, triggers platelet activation by stimulating granule secretion and releasing ATP, which then regulates endothelial junction disruption. Furthermore, adhesion molecules on platelets, such as integrins, selectins, and glycoproteins, enable them to protect CTCs from physical stress in the bloodstream. Mechanistically, activated platelets adhere to the surface of CTCs using a molecular bridge between GPIIb-IIIa and fibrinogen, providing protection against shear stress and evading immune cells [115, 116].

CTCs must also resist anoikis, a form of programmed cell death triggered by the loss of extracellular matrix (ECM) attachment, to successfully disseminate and establish metastatic colonies. Anoikis serves as a natural barrier to prevent aberrant cell detachment and proliferation in inappropriate locations. It involves a cascade of molecular events, including caspase activation, signaling pathway disruption, and DNA fragmentation, ultimately leading to cell death [117]. Platelets, however, aid tumor cells in evading anoikis and promoting metastasis. Mechanistically, autotaxin (ATX), stored in the α-granules of resting platelets, is released during tumor cell-induced platelet aggregation. ATX converts lysophosphatidylcholine into lysophosphatidic acid (LPA), which binds to LPAR-1 on CTCs, inducing anoikis resistance via the RhoA/YAP-1 signaling pathway [68]. In ovarian cancer, platelets further enhance survival by inducing RhoA-MYPT1-PP1-mediated dephosphorylation of YAP1. Dephosphorylated YAP1 translocates to the nucleus, driving the expression of pro-survival genes and inhibiting apoptosis [118] (Fig. 1D).

Taken together, these findings underline the crucial role of platelets in protecting CTCs from the multiple challenges they face in the bloodstream. The interaction between platelets and CTCs not only facilitates their survival, but also enhances their ability to resist apoptosis and promote colonization of distant organs. This understanding not only expands our knowledge of the mechanisms underlying metastatic dissemination, but also opens new avenues for the development of therapeutic strategies aimed at disrupting platelet-CTCs interactions and, consequently, limiting cancer spread.

### Platelets contribute to the immune evasion of tumor cells

In addition to resisting anoikis, CTCs must evade the immune response. Activated platelets play a crucial role in this process by protecting CTCs from the host immune system, specifically by affecting NK cell cytotoxicity and inhibiting T cell immune responses [119] In order for platelets to provide this protective function, they must undergo activation, a process demonstrated in several studies [120].

Tumor cells from cancers such as breast, ovarian, and prostate have been shown to induce platelet activation, leading to platelet aggregation and the expression of key surface molecules, including glucocorticoid-induced TNF-related ligand (GITRL) and P-selectin [121]. GITRL binds to its receptor on NK cells, inhibiting interferon-gamma (IFN-γ) secretion, impairing NK cell activation, and reducing their lytic capacity [122]. Subsequent studies have supported this mechanism, confirming its role in immune evasion [123–125]. Similarly, P-selectin binds to its receptor on the CTCs membrane, facilitating platelet aggregation around the tumor cell. This event not only protects the CTCs from NK cell recognition but also shields them from the shear forces of blood flow [126].

Activated platelets release soluble molecules that contribute to an immunosuppressive microenvironment. For example, TGF-β downregulates the NK cell group 2D (NKG2D) immunoreceptor, thereby reducing lytic activity and IFN-γ secretion, weakening tumor cell recognition and elimination [121, 124]. Studies have shown that platelets are a major source of TGF-β in the TME, negatively impacting T cell activity in colon cancer and melanoma [127, 128]. Additionally, platelets express glycoprotein A repetitions predominant (GARP), a glycoprotein that increases upon activation by various agonists or tumor cells. GARP can convert latent TGF-β from non-platelet sources into active TGF-β, further promoting an immunosuppressive microenvironment [127].

Tumor cell-platelet interactions also trigger the release of MICA and MICB from the tumor cell membrane into the TME. MICA and MICB are ligands that bind to the immunoreceptor NKG2D, blocking NK cell activation and altering NKG2D expression [129]. In this interaction, platelets can also transfer MHC class I (MHC-I) proteins to the tumor cell membrane through direct contact, providing additional protection against NK cells, as these cells cannot recognize tumor cells [119, 124].

Platelets also contribute to the recruitment of granulocytes, such as neutrophils, to the metastatic site. Activated platelets bound to cancer cells secrete CXCL4, CXCL5, and CXCL7, as well as mucins, which attract neutrophils from the bloodstream. This process is mediated by the interaction between platelet P-selectin and its ligand PSGL-1 on the neutrophil membrane, promoting their activation [130]. The mutual activation of platelets and neutrophils results in the formation of platelet-neutrophil complexes and the release of neutrophil extracellular traps (NETs), which contain proteases such as cathepsin G, elastase, and myeloperoxidase. These enzymes further activate platelets and enhance the coagulation cascade, facilitating tumor progression and evading immune surveillance [131, 132] (Fig. 1D).

Moreover, the importance of platelets in CTCs protection and survival has been confirmed by several studies. For example, thrombocytopenia, caused by platelet depletion or defective platelet production, significantly enhances NK cell-mediated killing of CTCs both in vitro and in vivo [133]. Depletion of platelets with an antiplatelet antibody increases the number of apoptotic cells, suggesting that platelets are crucial for CTCs survival [118].

In summary, platelets play a multifaceted role in CTCs protection by physically shielding them, creating an immunosuppressive microenvironment, and interacting with other components of the immune system, such as neutrophils. These interactions highlight the significant contribution of platelets to CTCs immune evasion and facilitate metastasis development.

### Platelets promote adhesion to the endothelium and the extravasation of tumor cells

CTCs that have survived due to platelet protection need to extravasate in order to establish metastatic dissemination. Platelets and their adhesion receptors facilitate both adhesion and extravasation of CTCs [124]. In particular, activated platelets form a complex with CTCs and act as a bridge to bind tumor cells to the endothelium via P-selectin [124, 134]. Then, P-selectin and integrin αIIbβ3 promote further recruitment of tumor cells, enhancing adhesion to the endothelium [135].

In addition, some molecules released by platelets contribute to tumor cell adhesion to the endothelium. The interaction between platelets and melanoma cells stimulates the release of acid sphingomyelinase (ASM) from platelets, which induces the generation of ceramides in the tumor cell membrane. These ceramides reorganize and cluster α5β1 integrins on tumor cells, facilitating their adhesion to the endothelium [136]. Similarly, platelet-derived microvesicles can transfer integrins such as CD41 to tumor cells, increasing their adhesion to fibrinogen and subsequently to the endothelium [77].

Endothelial activation is crucial for platelet binding to its surface. This activation can be induced by tumor cells or activated platelets, through the release of microvesicles and the interaction through the receptor-ligand CD40-CD154 [137–139]. As a result, endothelial cells express adhesion molecules such as E-selectin, P-selectin, VCAM-1 and ICAM-1, which facilitate the attachment of tumor cells and the recruitment of leukocytes. This activation stimulates the release of IL-6, tissue factor, CCL2 and CCL5 [140, 141]. These interactions are essential for the adhesion of tumor cells to the endothelium and the subsequent development of metastasis.

Once CTCs adhere to the endothelium, platelets further facilitate the metastatic process. The extracellular matrix is proteolyzed by metalloproteinases secreted by activated platelets, leukocytes and tumor cells, which promotes matrix degradation [77, 142, 143]. Furthermore, activated platelets release thromboxane A2 and ATP from dense granules, inducing endothelial cell retraction, in turn, ATP binds to the P2Y2 receptor on endothelial cells, increasing endothelial permeability and facilitating CTCs extravasation [120, 144] (Fig. 1E).

### Platelets contribute to the development of chemoresistance

Chemoresistance represents one of the most important challenges in oncology, limiting the efficacy of multiple therapeutic strategies. This phenomenon may be due to various mechanisms intrinsic and extrinsic to tumor cells. Nowadays, platelets have been recognized as playing a crucial role in promoting chemoresistance by facilitating cell survival, evasion of apoptosis and the creation of a tumor-protective microenvironment.

Several studies have demonstrated the impact of platelets on chemoresistance across various cancer types. In pancreatic cancer, platelet release promoted the upregulation of p-Erk and p-Akt, both involved in cell survival signaling. Moreover, through the regulation of Slug, platelet release decreased the expression of human equilibrative nucleoside transporter 1 (hENT1), responsible for gemcitabine uptake, and increased the expression of cytidine deaminase (CDD), which inactivates this chemotherapeutic agent. These alterations not only reduce the efficacy of gemcitabine, but also promote the proliferation of tumor cells even under treatment [145]. Similarly, in ovarian cancer, platelet release promotes the formation of tumor spheroids with high expression of tumor stem cell markers such as CD133 and aldehyde dehydrogenase (ALDH), which show resistance to cell death induced by cisplatin, carboplatin, and paclitaxel [146]. Additionally, conditioned medium from platelets and colon cancer cells also promotes resistance to oxaliplatin treatment [147].

PMPs also promote resistance to daunorubicin-induced cell death in acute myeloid leukemia cells by reducing the expression of the proapoptotic protein PUMA, inhibiting the activation of caspase-8 and caspase-9, and decreasing daunorubicin-induced DNA damage [148]. Comparable mechanisms have been identified in ovarian and colon cancer cells, where platelets increased cell survival in response to 5-fluorouracil and paclitaxel by upregulating antiapoptotic genes and downregulating proapoptotic genes. Furthermore, they activated key proteins involved in DNA repair, such as phospho-BRCA, Chk1, Mre11, and p95/Nbs1, while upregulating MAPK pathways, including p38 and JNK-p54 [149]. These findings highlight the crucial role of platelets in regulating apoptosis and DNA repair.

The relevance of platelets in chemoresistance is not limited to in vitro studies. In an orthotopic model of ovarian cancer, treatment with antiplatelet antibodies or docetaxel significantly reduced tumor growth, while the combination of both treatments showed synergistic effects. In contrast, the increase in the number of platelets per transfusion favored tumor growth and decreased the effectiveness of docetaxel, reinforcing the pro-tumoral role of platelets and their influence on chemotherapy resistance [150]. In fact, in patients with rectal cancer who presented thrombocytosis before treatment, a lower 3-year disease-free survival rate and a lower overall survival were reported compared to those with normal platelet counts, establishing thrombocytosis as a potential negative predictive marker for chemotherapy response [151].

Platelet infiltration into the tumor microenvironment has also been implicated in chemoresistance. In breast cancer, platelets surrounding primary tumor cells were linked to reduced sensitivity to neoadjuvant chemotherapy [152]. Whereas, in gastric cancer, histological evidence of platelet aggregation within the tumor stroma correlated with significantly higher rates of treatment resistance [153]. These findings suggest that activated platelets contribute to creating a microenvironment that shields cancer cells and impairs the effectiveness of chemotherapy (Fig. 1F).

Taken together, the mechanisms described highlight the critical role of platelets in promoting a tumor microenvironment favorable to chemoresistance. Future research should focus on identifying strategies that limit the interaction between platelets and tumor cells, which could enhance the efficacy of oncological treatments and improve clinical outcomes.

### Mitochondrial transfer from platelets to tumor cells

Platelets have also been implicated in metabolic alterations in cancer. Recently, a mechanism has been reported by which platelets can transfer mitochondria to tumor cells from different types of cancer, leading to changes in their metabolism and malignancy [154]. This suggests a novel mechanism by which platelets can contribute to the metastasis process.

Mitochondrial transfer is a physiological process to maintain homeostasis, however, it is also observed in pathophysiological processes such as cancer, this transfer can occur through nanotube formation, cell fusion, gap junctions, and the exchange of extracellular microvesicles [155]. PMPs are the main mechanism reported by which platelets can transfer mitochondria to tumor cells [156]. In 2023, Gharib et al., reported that PMPs are captured by leukemia tumor cells, leading to metabolic reprogramming and improving neoplastic characteristics [76]. Similarly, Cecereda et al., reported that MDA-MB-231, a breast cancer cell line, is capable of acquiring mitochondria from platelet-derived microparticles, impacting cellular metabolism and improving neoplastic characteristics [69]. Interestingly, it has been observed that the permeability of recipient cells to microparticles internalization varies among cancer cell types. In breast cancer, Veilleux et al., evaluated the acquisition of mitochondria from PMPs in three breast cell types, finding differences in the internalization of mitochondria from PMPs into the cell, confirming that mitochondria internalization led to metabolic changes and a diversity of neoplastic characteristics [157]. Mitochondrial transfer has also been evaluated in vivo and efforts have been made to attempt to elucidate a transfer mechanism. In this regard, Zhang et al. reported that cancer cells are reprogrammed to a metastatic state through the acquisition of platelet mitochondria via the PINK1/Parkin-Mfn2 pathway. The transferred platelet mitochondria drove a metabolic shift and ROS reduction in cancer cells both in vitro and in vivo assays [158] (Fig. 1G). Following these observations, targeting platelet mitochondria has been suggested as a potential treatment for metastases, although further studies are required for this development.

### The potential of platelets as therapeutic vehicles in cancer

Conventional chemotherapy and immunotherapy can show limited success and relevant side effects. Current research aims to develop drug delivery systems that can specifically accumulate in tumor tissues, can selectively recognize and enter cancer cells, and can precisely target subcellular sites. With the above in mind, it is interesting to consider the use of platelets as therapeutic vehicles since they infiltrate the TME, can reach specific receptors to adhere to cancer cells, and exchange microvesicles. Interestingly, platelets have been used as vehicles to administer drugs loading therapeutic compounds directly into their cytoplasm using techniques such as electroporation or membrane detergents [159–162]. For example, loading doxorubicin into platelets is effective in accumulating the drug and delivering it directly to tumor cells. In vivo studies showed that this approach induced tumor cell apoptosis, reduced tumor size, and caused fewer side effects. Activation of platelets by tumor cells facilitated drug release within the target cells, and an acidic environment also helped release doxorubicin more effectively. This method using loaded platelets proved to be more potent than doxorubicin alone, but without the associated toxic effects [159, 162, 163].

Photothermal therapy has been investigated in combination with platelets. Platelets can act as carriers for photothermal agents and enhance drug targeting within the TME. When combined with localized laser irradiation, this method has been shown to inhibit tumor growth [164, 165]. Additionally, the incorporation of inactivated viral particles into platelets has been explored to stimulate antitumor immunity. In a murine model of melanoma, platelets effectively directed these particles to the tumor, reducing tumor growth [166]. Coating nanoparticles with platelet membranes has emerged as a method to address challenges in drug delivery. Platelet membrane-coated nanoparticles have proven effective in delivering doxorubicin, crossing the blood–brain barrier, and targeting surgical margins in residual glioblastoma multiforme [167]. They have also been beneficial in reducing postoperative recurrence in early hepatocellular carcinoma [160], as well as in enhancing in vivo immune responses and reducing lung metastasis [168]. Furthermore, in sonodynamic therapy, an emerging technique that uses ultrasound to activate targeted agents and induce antitumor effects, coating therapeutic agents with platelet membranes has been shown to increase their circulation time as well as improve tumor targeting [169].

Platelet genetic engineering has also emerged as an innovative strategy in cancer treatment. This technique uses the inherent abilities of platelets to deliver specific therapies directly to tumor cells. One promising approach involves the genetic modification of platelets to express surface-bound tumor necrosis factor-related apoptosis-inducing ligand (TRAIL), a cytokine known to induce apoptosis specifically in tumor cells, which has shown efficacy in inhibiting tumor growth and metastasis [170] (Fig. 2B).

In summary, platelet-based drug delivery systems offer significant advantages, such as increased biocompatibility, reduced adverse effects, and prolonged drug circulation time. Because platelets can be isolated in large quantities and are frequently transfused in clinical settings, they offer a promising platform for these systems. Furthermore, combining classic chemotherapy or gene therapy techniques, such as nucleic acids, aptamers, or siRNAs, with platelets in cancer treatment promises to improve therapeutics into a specific effective, and even personalized field in the fight against cancer.

### Future directions and clinical implications of the role of platelets in cancer

Despite advances in understanding the role of platelets in cancer, significant challenges remain that limit a full characterization of the mechanisms involved. For example, current studies lack standardized protocols to analyze platelet interaction with tumor cells, including variations in isolation methods, verification of their activation, and accurate determination of their number or concentration in experiments, which hinders reproducibility and comparison of results. While two-dimensional models have been useful for studying processes such as EMT, the use of three-dimensional systems, organoids, or microfluidic platforms that better reproduce the tumor environment could offer a more accurate and physiologically relevant view of the role of platelets in cancer. Furthermore, it is crucial to advance the use of in vivo models to investigate key processes such as intravasation, extravasation, angiogenesis, and metastasis formation. These models are essential not only to validate findings obtained in vitro, but also to identify new therapeutic strategies aimed at mitigating tumor progression. Overcoming these limitations will allow us to explore the molecular mechanisms that link platelets to cancer progression, opening the door to more precise and effective therapeutic interventions.

On the other hand, although there are already clinical trials analyzing the use of antiplatelet agents in cancer patients, the number of studies is limited and the results are not yet conclusive. More robust clinical trials with a greater focus on modulating platelet-tumor interaction are needed to determine whether this strategy has a significant clinical impact on cancer progression and whether it can be used as an effective complementary therapy.

### Tumor-educated platelets

Timely diagnosis remains a major challenge in cancer care. Addressing this challenge requires the development of screening and early detection tests, along with effective follow-up methods. These strategies are crucial for optimizing therapy effectiveness and reducing cancer-related mortality. Currently, oncology research focuses on analyzing various biological fluids rather than whole tissues to identify tumor-derived components, a technique known as liquid biopsy. The term 'liquid biopsy' refers to the collection of tumor-derived biomarkers, including circulating tumor DNA (ctDNA), circulating RNA (cfRNA), CTCs, tumor-derived extracellular vesicles, and tumor-educated platelets (TEPs), found in blood and other body fluids such as urine, saliva, feces, or cerebrospinal fluid [171, 172]. Liquid biopsies have gained prominence due to their minimal invasiveness compared to traditional tissue biopsies, enabling repeated sampling throughout treatment. This provides real-time, accurate information on both the tumor and its microenvironment, as well as the detection of metastatic sites. Furthermore, liquid biopsies offer the potential for molecular profiling, which aids in identifying relevant biomarkers and personalizing cancer therapy [171, 173]. Together these highlights, platelets as a promising biosource for liquid biopsies.

Despite lacking a nucleus, platelets have been reported to inherit a functional spliceosome, small nuclear ribonucleic acids (snRNA), and pre-mRNA from their precursor cells, the megakaryocytes. In response to external stimuli, they are capable of performing pre-mRNA splicing, even without a nucleus, allowing for a level of gene expression that was previously thought to be impossible [174]. In addition, platelets contain ribosomes, enabling them to carry out protein synthesis. This ability to synthesize proteins de novo through regulated mRNA translation is particularly important in the context of physiological and pathological stimuli. Moreover, microRNAs [175], long non-coding RNAs (lncRNAs) [176], and circular RNAs (circRNAs) [177] within platelets contribute to post-transcriptional regulation, further expanding their functional capabilities.

These molecular characteristics make platelets particularly sensitive to environmental changes, such as those induced by cancer. In fact, recent studies have shown that platelets exposed to tumors undergo significant alterations in their RNA and protein profiles—a phenomenon described as TEPs [178].

**Fig. 3:**
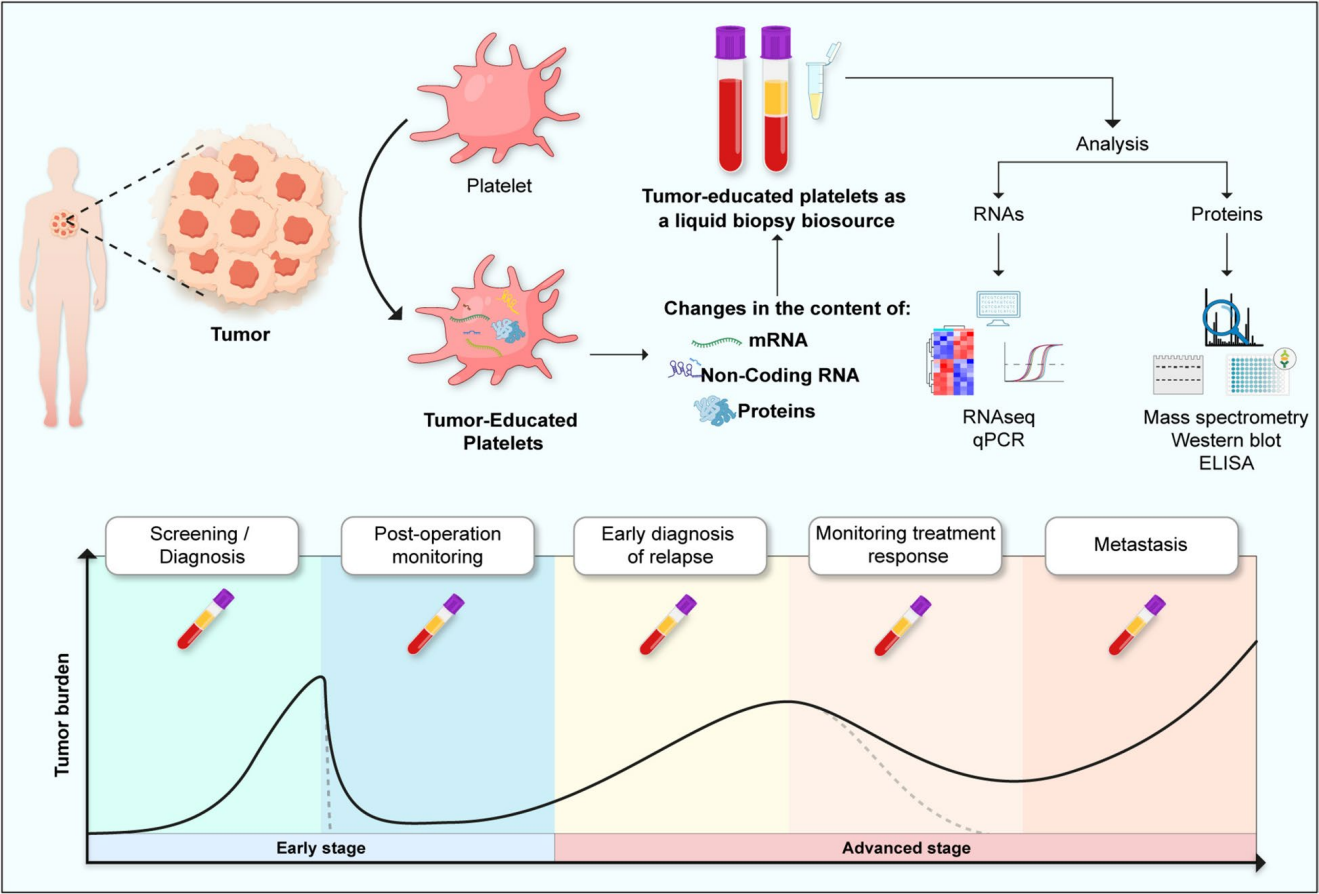
Tumor-educated platelets (TEPs) represent a promising source of biomarkers in liquid biopsies for cancer diagnosis and monitoring. Through the analysis of messenger RNA (mRNA), non-coding RNA (ncRNA) and proteins, TEPs allow the detection of molecular alterations associated with tumor presence. These characteristics make TEPs useful not only in the diagnosis of early disease, but also in monitoring progression, response to treatment, detection of residual disease and identification of metastases, thus facilitating personalized medicine and improving outcomes in cancer patients

This "education" process occurs through several mechanisms: the incorporation of tumor-derived biomolecules (proteins, nucleic acids, and vesicles), the regulation of specific mRNA splicing events, and changes in the megakaryocytes that produce platelets. As a result, the contents of platelets change in the presence of a tumor, leading to a dynamic profile of their RNA and proteins. This change makes them useful for liquid biopsies [179–181]. By using this changing profile, TEPs provide a new and flexible source for cancer diagnosis and monitoring. They can reflect real-time changes in the tumor, making them a valuable tool for liquid biopsies and potentially transforming personalized cancer care (Fig. 3).

### The mRNA profile is different in TEPs

Even though platelets contain less mRNA than other blood cells, next-generation sequencing studies have identified an mRNA repertoire of 3,000 to 6,000 transcripts [182, 183]. This RNA diversity underscores the potential of using platelets in liquid biopsies. In fact, some authors have identified distinct gene expression profiles in platelets from both healthy individuals and patients with different types of cancer, including breast, lung, colorectal, glioblastoma, pancreatic, and hepatobiliary cancers [178]. Thus, transcriptome changes can be used to distinctively classify cancer patients. The transcriptome of TEPs has shown a 96% accuracy in distinguishing between patients with localized or metastatic tumors and healthy individuals, correctly identifying the primary tumor location in 71% of cases [178]. Moreover, recent research has shown that TEPs can distinguish between patients with early- and advanced-stage cancer in various tumor types [184].

TEPs have been used in biomarker identification. For example, in esophageal squamous cell carcinoma, a three-gene molecular signature was identified, demonstrating a sensitivity of 87.5% and a specificity of 81.1%, being superior to other existing biomarkers [185]. In colorectal cancer, these platelets demonstrated a significant diagnostic value, with an area under the curve (AUC) of 0.92, superior to traditional serum biomarkers such as carcinoembryonic antigen and CA-19–9 [186]. Moreover, TEPs displayed distinct alterations in patients with sarcoma, enabling their differentiation from both healthy donors and patients in remission [187]. In a study by Huber et al., the transcriptional profile of TEPs was compared to that of platelets from healthy donors, identifying the alpha 1 chain of type I collagen (COL1A1) as a potential biomarker for head and neck squamous cell carcinoma. Interestingly, in this study, TEPs showed 426 differentially expressed RNAs compared to platelets from healthy donors, of which 254 were tumor-related. These findings suggest that tumors not only transfer RNA to platelets but also induce a unique transcriptional profile in response to tumor presence [188].

Advanced computational approaches have further enhanced biomarker identification. Particle swarm optimization (PSO), an advanced algorithm, has enhanced the efficiency of selecting gene panels as biomarkers from RNA sequencing data of TEPs enabling an accurate distinction between patients with non-small cell lung cancer, healthy individuals, and those with non-cancerous inflammatory conditions [189]. Similarly, the deep learning image-based classifier imPlatelet, which converts platelet RNA sequencing data into images where each pixel represents the expression level of a specific gene, can identify cancer cases with high accuracy [190, 191]. For example, this classifier differentiated between patients with endometrial cancer and healthy donors with an AUC of 97.5% and between patients with endometrial cancer and those with benign gynecological conditions, achieving an AUC of 84.1% [192].

Although machine learning algorithms in bioinformatics hold great potential for cancer detection, their limitations, including the high demand for computing capacity and associated costs, highlight the need to explore biomarkers in TEPs using more accessible molecular techniques. Identifying RNA biomarkers from platelet RNA sequencing, followed by validation through quantitative real-time PCR (qPCR), is a viable approach. In this context, several studies have proposed specific biomarkers. For example, in the study by Xing, integrin alpha 2b (ITGA2B) expression was identified as a promising biomarker for non-small cell lung cancer with an AUC of 0.92 [193]. Similarly, TIMP1 has been proposed as a biomarker in colorectal cancer (AUC 0.95) and tropomyosin 3 (TPM3) in breast cancer (AUC 0.97) [194, 195]. Moreover, given the exchange of RNA between platelets and tumor cells, searching for known cancer biomarkers in the platelets of cancer patients presents a viable strategy. In this regard, transcripts of prostate cancer biomarkers, including kallikrein-related peptidase-2 and −3 (KLK2, KLK3), folate hydrolase 1 (FOLH1), and neuropeptide-Y (NPY), were found exclusively in the platelet fraction of cancer patients and were absent in healthy controls [196]. These findings highlight the potential of combining bioinformatics tools with molecular techniques to refine platelet biomarker identification, ultimately advancing early cancer detection and monitoring. A summary of studies on mRNA changes in TEPs is presented in Table 1.

**Table 1:** Clinical data related to the diagnosis of various cancer types utilizing TEPs

Cancer type	Biomarkers	Clinical Diagnostic Accuracy	References
Esophageal squamous cell carcinoma	*RID1A, GTF2H2*, and *PRKRIR*	Sensitivity of 87.5% and specificity of 81.1%	[185]
Colorectal cancer	TEP RNA profiling	AUC of 0.92	[186]
	*TIMP1*	AUC of 0.95	[194]
Endometrial cancer	*imPlatelet Classifier*	AUC of 0.97	[192]
Non-small cell lung cancer	*ITGA2B*	AUC of 0.92	[193]
Breast cancer	*TPM3*	AUC of 0.97	[195]
Pan-Cancer	TEP transcriptome	AUC of 0.98	[178]
Pan-Cancer	*Pan-cancer-platelet* RNA biomarker panel	AUC 0.91	[184]

### Changes in the non-coding RNA profile of TEPs

In addition to mRNAs, platelets contain a wide repertoire of non-coding RNAs, such as microRNAs, circRNAs, and lncRNAs, whose potential in liquid biopsies has been recently explored. MicroRNAs, short sequences of 18 to 25 nucleotides, regulate gene expression at the post-transcriptional level by interfering with protein translation or promoting mRNA degradation through their binding to untranslated regions (UTRs) of mRNA. These microRNAs have been widely studied as biomarkers for the diagnosis, prognosis, and treatment of cancer, with specific profiles identified in various cancer types [197].

In platelets, microRNAs are abundantly expressed, and platelets contain key enzymes such as Dicer and Argonaute 2 (Ago2), both essential for miRNA maturation. This suggests that, like mRNAs, platelet miRNA content is dynamic and clinically relevant for disease diagnosis. For example, in nasopharyngeal carcinoma (NPC), PET miR-34c-3p and miR-18a-5p have been observed to be significantly higher in NPC patients compared to healthy subjects, underscoring their clinical value for diagnosis [198]. In pancreatic cancer, differential microRNAs expression has also been detected in platelets, attributed to the horizontal transfer of microRNAs between tumor cells and platelets [199]. In this context, miR-223 levels in platelets and PMPs from non-small cell lung cancer (NSCLC) patients were higher than in healthy subjects. These microparticles efficiently transferred miR-223 to lung cancer cells, promoting cell invasion by targeting EPB41L3 [200]. In liver cancer, microRNAs miR-495-3p and miR-1293 also showed differential expression, highlighting their potential for early and accurate detection of the disease [201].

LncRNAs are RNA molecules longer than 200 nucleotides that regulate gene expression through various mechanisms, such as epigenetic modifications, intracellular transport, RNA splicing, and transcriptional regulation [202]. In the context of tumors, lncRNAs can influence cancer progression, and changes in their expression can provide valuable information for cancer diagnosis [203]. In platelets from colorectal cancer patients, Ye et al., identified 128 differentially expressed lncRNAs including upregulation of LNCAROD, SNHG20, LINC00534, and TSPOAP-AS1 also found elevated in serum and tumor-adjacent tissues. Expression levels of LNCAROD and TSPOAP-AS1 correlated significantly with cancer stage and tumor location [204, 205]. On the other hand, in non-small cell lung cancer (NSCLC), decreased expression of the lncRNAs MAGI2 antisense RNA 3 (MAGI2-AS3) and ZNFX1 antisense RNA 1 (ZFAS1) was reported in both plasma and platelets. Reduced MAGI2-AS3 expression was associated with cancer TNM stage, lymph node metastasis, and distant metastasis, while ZFAS1 alone correlated with TNM stage. The combination of MAGI2-AS3 and ZFAS1 demonstrated a superior ability to discriminate between NSCLC and controls, suggesting that these lncRNAs could serve as biomarkers for NSCLC diagnosis [206].

In lung cancer, the TEPs lncRNAs linc-GTF2H2-1 and RP3-466P17.2 were downregulated, while lnc-ST8SIA4-12 was upregulated, and implemented in combination of these three lncRNAs improved diagnostic accuracy. Moreover, integrating linc-GTF2H2-1 with specific markers like CEA, Cyfra21-1, or NSE helped to distinguish between advanced and early disease stages [207]. In nasopharyngeal carcinoma, a significant reduction in lncRNA ROR expression was observed in the platelets of cancer patients. However, no changes were found in its plasma levels, suggesting the alteration is platelet-specific [208]. Table 2 summarizes the most recent studies.

**Table 2:** Changes in the platelet transcriptome associated with different types of cancer

RNA type	Cancer type	Expression in platelets	References
microRNAs	Nasopharyngeal carcinoma	↑miR-34c-3p	[198]
		↑ miR-18a-5p	
	Non-small cell lung cancer	↑ miR-223	[200]
	Liver cancer	↓ miR-495-3p	[201]
		↑miR-1293	
lncRNAs	Colorectal cancer	↑ LNCAROD	[204, 205]
		↑ SNHG20	
		↑ LINC00534	
		↑ TSPOAP-AS1	
		↑ CCAT1	
		↑ HOTTIP	
	Non-small cell lung cancer	↓ MAGI2-AS3	[206]
		↓ ZFAS1	
	Lung cancer	↓ linc-GTF2H2-1	[207]
		↓ RP3-466P17.2	
		↑ lnc-ST8SIA4-12	
	Nasopharyngeal carcinoma	↓ ROR	[208]

↑: Upregulated; ↓: Downregulated

In summary, platelet-derived non-coding RNAs, including microRNAs and lncRNAs, are emerging as promising biomarkers in oncology. Their ability to reflect tumor-specific molecular changes and their potential to improve diagnostic and prognostic accuracy underscore the importance of further investigating their role in liquid biopsies. These advances could greatly contribute to developing more effective and less invasive tools for cancer detection and monitoring.

### Changes in the proteome of TEPs

Platelets contain a diverse array of proteins, such as growth factors, chemokines and proteases, which play a crucial role in regulating hemostasis. These proteins are first synthesized by megakaryocytes and transferred to platelets. Moreover, platelets can autonomously synthesize proteins by maturing and processing mRNA in response to external stimuli and can selectively absorb proteins from the microcirculation, making them a dynamic protein reservoir [209].

In vivo studies have shown that tumors release various proteins, leading to elevated levels in circulation. Interestingly, these tumor-derived proteins tend to accumulate in higher concentrations in platelets. It has been proposed that platelets could mediate communication between the primary tumor and metastatic sites through the transport of these molecules [210, 211]. Additionally, research in mouse models has shown that the growth factor content in circulating platelets changes during the early stages of tumor development, reinforcing the dynamic role of platelets in cancer progression [29, 209, 212].

These changes in platelet proteins have also been suggested as a potential source of biomarkers. In patients with lung and pancreatic cancer, the platelet proteome significantly differs from that of healthy individuals. Interestingly, after surgical tumor resection, the platelet proteome in these patients normalized [213]. A subsequent study by the same group confirmed these findings and identified overlapping proteins across other cancer types, highlighting RNF213, CTSG, PGLYRP1, RPL8, S100A8, S100A9, GPX1, and TNS1 as promising biomarkers for cancer detection [214]. Another study found elevated levels of VEGF, PF4, and PDGF in platelets of colorectal cancer patients, demonstrating their utility in distinguishing healthy individuals from those with cancer [215]. Interestingly, these same proteins were altered in other cancers, like lung and pancreatic cancer, suggesting that platelet protein changes may be more related to the presence of cancer in general rather than a specific tumor type [216]. These alterations in the platelet proteome offer a promising avenue for developing biomarkers useful in cancer detection and monitoring, regardless of tumor type. This underscores the potential of platelets as a valuable and versatile diagnostic tool and biomarker in oncology. The main studies on changes in the protein profiles of tumor-educated platelets are summarized in Table 3.

**Table 3:** Changes in the platelet proteome associated with different types of cancer

Cancer type	Protein expression in platelets	References
Lung Cancer, Pancreas cancer	↑ RELN, LRSAM1, RPS27L, ENY2, CTSG and S100A9	[213]
	↓ ATP6Ap1	
Colon, rectal, anal, esophageal, gastric, tongue and primitive neuroectodermal cancer	↑ RNF213, CTSG, PGLYRP1, S100A8, S100A9 and RPL8	[214]
	↓ GPX1, TNS1	
Colorectal cancer	↑ VEGF, PF4 and PDGF	[215]
Lung cancer	↑ VEGF and PDGF	[216]
	↓ PF4, CTAPIII and TSP-1	
Pancreas cancer	↑ VEGF	

↑: Upregulated; ↓: Downregulated

### Future directions and clinical implications of tumor educated platelets

Currently, TEPs hold great promise as liquid biopsies; however, despite advancements, significant challenges remain for their clinical implementation. One of the main obstacles is the lack of knowledge about the precise mechanisms by which platelets are “educated” in the tumor context. Although it is well-documented that platelets alter their profile in response to the tumor, the exact mechanisms underlying this process remain unclear. It is essential to determine whether this change results from an exchange of molecules or microvesicles between the tumor and platelets, internal platelet synthesis, the absorption of tumor derivatives, the interaction with CTCs, or a combination of these mechanisms. Moreover, a less explored hypothesis suggests that platelets might be educated through megakaryocytes. The lack of knowledge about these mechanisms underscores the need to investigate how platelets acquire their characteristics in the tumor context. Likewise, the role of platelets derived from pulmonary megakaryocytes remains an underexplored area and deserves further attention.

A key area for improvement is refining methods to identify specific TEP biomarkers, such as mRNA, ncRNAs, and proteins. Current methods of platelet isolation, such as differential centrifugation, allow efficient extraction but do not prevent contamination with other blood cells, which decreases platelet specificity and interferes with biomarker selection. Furthermore, the tendency of platelets to activate favors a change in the content of their molecules, which makes biomarker identification difficult. Addressing these challenges requires developing standardized and optimized methods for platelet isolation and purification. Furthermore, it is essential to analyze TEPs to identify and validate unique molecular signatures in different types of cancer, in addition to conducting large-scale clinical trials that evaluate their potential, ensuring adequate selection of patients according to their clinical variables, as well as the choice of an optimal control group to facilitate biomarker identification. It would also be beneficial to explore other forms of platelet transcriptome analysis, such as single-cell RNA sequencing, which would allow the identification of distinct platelet populations within TEPs, facilitating the characterization of their heterogeneity and the identification of molecular signatures associated with different types of cancer.

Although TEPs represent a promising tool, their full potential can only be achieved by overcoming these challenges. Integrating advanced RNA and protein analysis techniques with traditional molecular approaches could pave the way for their clinical application, enhancing cancer diagnosis, monitoring, and treatment. Addressing these challenges will not only consolidate platelets as a reliable tool in oncology, but could also open new opportunities for their application in other diseases.

## Conclusions

This review underscores the critical role of platelets in cancer progression. Over the past decades, significant advances have been made in understanding platelet biology and their diverse interactions with tumor cells and their broader impact on oncogenic processes. However, despite these insights, translating this knowledge into tangible clinical breakthroughs remains a challenge. Within this review, we have explored key themes that hold promise for advancing cancer diagnostics and therapeutics. In particular, biomarkers derived from TEPs have emerged as a valuable biosource for liquid biopsies, offering a non-invasive approach to cancer detection, diagnosis, and monitoring. TEP-based biomarkers could allow earlier cancer diagnosis, reduce costs by guiding molecularly targeted treatments, enhance patient convenience, and ultimately support more precise and informed oncological decision-making. Additionally, targeting platelet-mediated mechanisms presents an opportunity for novel therapeutic strategies in the era of precision medicine. Future research should prioritize bridging the gap between fundamental discoveries and clinical applications. This includes developing targeted approaches to modulate platelet activity in cancer while preserving their essential hemostatic functions. By leveraging the expanding knowledge of platelet-tumor interactions, the field is poised to uncover innovative diagnostic and therapeutic solutions that could significantly improve patient outcomes.

## Data Availability

No datasets were generated or analysed during the current study.

## Abbreviations

ADP: Adenosine di-phosphate
Ago2: Argonaute 2
ALDH: Aldehyde dehydrogenase
ASM: Acid sphingomyelinase
ATP: Adenosine tri-phosphate
ATX: Autotaxin
AUC: Area under the curve
BMDCs: Bone marrow-derived cells
CDD: Cytidine deaminase
cfRNA: Circulating RNA
circRNAs: Circular RNAs
CTCs: Circulating tumor cells
ctDNA: Circulating tumor DNA
EGF: Epidermal growth factor
EMT: Epithelial-mesenchymal transition
FAK: Focal adhesion kinase
FGF: Fibroblast growth factor
FOLH1: Folate hydrolase 1
GARP: Glycoprotein A repetitions predominant
GITRL: TNF-related ligand
hENT1: Human equilibrative nucleoside transporter 1
HRG: Histidine-rich glycoprotein
IGF: Insulin-like growth factor
IGFBP: Insulin-like growth factor binding protein
ILs: Interleukins
ITGA2B: Integrin alpha 2b
KLF6: Krüppel-like factor 6
KLK2, KLK3: Kallikrein-related peptidase-2 and -3
lncRNAs: Long non-coding RNAs
LPA: Lysophosphatidic acid
LRG1: Leucine-rich alpha-2-glycoprotein 1
MAGI2-AS3: MAGI2 antisense RNA 3
MHC-I: Major Histocompatibility Complexclass I
NETs: Neutrophil extracellular traps
NKG2D: NK cell group 2D
NPC: Nasopharyngeal carcinoma
NPY: Neuropeptide-Y
NSCLC: Non-small cell lung cancer
OLFM4: Olfactomedin 4
PDAF: Platelet-derived angiogenesis factor
PDGF: Platelet-derived growth factor
PEVs: Platelet-derived extracellular vesicles
PMPs: Platelet-derived microparticles
PRP: Platelet-rich plasma
PSO: Particle swarm optimization
qPCR: Real-time PCR
snRNA: Small nuclear ribonucleic acids
TBK1: TANK-binding kinase 1
TCIPA: Tumor cell-induced platelet aggregation
TEPs: Tumor-educated platelets
TGF: Transforming growth factor
TME: Tumor microenvironment
TPM3: Tropomyosin 3
TxA2: Thromboxane A2
UTRs: Untranslated regions
VEGF: Vascular endothelial growth factor
vWF: Von Willebrand factor
ZFAS1: ZNFX1 antisense RNA 1
